# Compartment-specific oxidative stress signatures in oral leukoplakia: a systematic review of local and systemic biomarkers

**DOI:** 10.3389/fphys.2026.1851447

**Published:** 2026-06-03

**Authors:** Cristina Estornut, Ulrikke Pauline Clark-Utvik, Nicla Flacco, Pilar Ribera, Germán Sánchez-Herrera, Martín Pérez-Leal

**Affiliations:** 1 Faculty of Health Sciences, Universidad Europea de Valencia, Valencia, Spain; 2 Centro Avanzado de Microbiología Aplicada, Universitat Politècnica de València, Valencia, Spain

**Keywords:** antioxidants, biomarkers, oral leukoplakia, oxidative stress, reactive oxygen species

## Abstract

**Introduction:**

Oral leukoplakia (OLK) is one of the most common potentially malignant disorders of the oral mucosa. Oxidative stress is increasingly recognized as a key factor in its pathogenesis and malignant transformation, but the consistency of biomarker alterations across biological compartments remains unclear.

**Aim:**

To evaluate compartment-specific oxidative stress alterations across local (tissue, saliva) and systemic (blood, plasma, serum) environments in OLK by synthesizing evidence on biomarker levels in these biological compartments.

**Materials and methods:**

A systematic search was conducted in PubMed, Web of Science and Scopus, following PRISMA guidelines. Studies were included if they compared oxidative stress biomarkers between OLK patients and healthy controls. Data were qualitatively synthesized, and study quality was assessed using the Newcastle-Ottawa Scale, ROBINS-I (Cochrane), and AXIS. The protocol was registered in the International Prospective Register of Systematic Reviews (PROSPERO) under registration number CRD42023438437.

**Results:**

Fifteen studies were included (n = 804). Oxidative damage markers such as malondialdehyde (MDA) and 8-OHdG were consistently elevated in OLK patients across tissue, serum, plasma, blood, and saliva. Enzymatic antioxidants such as GPx, SOD, and CAT were generally reduced, whereas tissue levels of glutathione (GSH) were elevated, suggesting a local compensatory response. Total antioxidant capacity (TAS/TAC) was decreased systemically. Notably, antioxidant responses differed across biological compartments, with increased tissue GSH contrasting with systemic depletion.

**Conclusion:**

The available evidence supports a consistent redox imbalance in OLK characterized by increased oxidative damage and impaired systemic antioxidant defenses. Salivary and serum biomarkers, particularly MDA and 8-OHdG, show promise as non-invasive indicators of oxidative stress and disease progression. However, methodological heterogeneity and small sample sizes limit clinical translation. Standardized protocols and longitudinal studies are needed to validate these biomarkers.

## 1 Introduction

Oral leukoplakia (OLK) is the most common potentially malignant lesion of the oral mucosa, defined as a predominantly white lesion of the oral mucosa that cannot be characterized as any other definable lesion (Axéll et al., 1996). It carries a variable risk of malignant transformation, generally ranging from 1% to 20%, depending on factors such as lesion size and persistence, anatomical location (particularly the tongue and floor of the mouth), clinical presentation, and, most importantly, the presence and severity of epithelial dysplasia. Recent reviews report an overall mean transformation rate of approximately 9–10%, although with considerable heterogeneity among studies (Imbesi Bellantoni et al., 2023; Maloney et al., 2024). Early identification of high-risk lesions is essential to prevent progression to oral squamous cell carcinoma (OSCC), an aggressive and prevalent head and neck malignancy (Imbesi Bellantoni et al., 2023).

Oxidative stress, defined as an imbalance between reactive oxygen species (ROS) and antioxidant defenses, has emerged as a key contributor to carcinogenesis (Sies, 2015; Čižmárová et al., 2022; Jomova et al., 2023). ROS can damage lipids, proteins, and DNA, promoting genomic instability and tumor development. The oral cavity is particularly vulnerable to oxidative damage due to chronic exposure to tobacco, alcohol, inflammation, and microbial agents (Scholtes and Giguère, 2021; Imbesi Bellantoni et al., 2023). In response, the body activates antioxidant defenses such as superoxide dismutase (SOD), catalase (CAT), glutathione peroxidase (GPx), and glutathione (GSH), alongside markers of oxidative damage like malondialdehyde (MDA) and 8-hydroxy-2’-deoxyguanosine (8-OHdG) (Korraah et al., 2012; Demirci-Çekiç et al., 2022).

However, findings regarding oxidative stress in OLK remain partially contradictory, and no clinically validated biomarkers currently exist to predict malignant transformation. Diagnosis still relies on exclusion and clinical experience, which can be challenging for less experienced clinicians (Axéll et al., 1996; Sies, 2015). The variability of biomarker expression between local and systemic compartments adds further complexity (Korraah et al., 2012; Čižmárová et al., 2022; Demirci-Çekiç et al., 2022). Identifying reliable and non-invasive redox biomarkers could improve early detection and risk stratification.

This review aims to synthesize current evidence on oxidative and antioxidant biomarker levels in patients with oral leukoplakia (OLK) compared with healthy controls, across different biological compartments (saliva, blood/serum, plasma, and tissue). The purpose is to explore patterns of redox imbalance associated with OLK and to identify which biomarkers show the most consistent alterations, without evaluating diagnostic accuracy or prognostic capacity.

## 2 Materials and methods

This systematic review was conducted in accordance with the PRISMA (Preferred Reporting Items for Systematic Reviews and Meta-Analyses) guidelines (Moher et al., 2010). The protocol was registered in the International Prospective Register of Systematic Reviews (PROSPERO) under registration number CRD42023438437.

Based on the objectives of this systematic review, the research question focuses on the following components:

- P (Population): Patients with OLK
- I (Intervention): Measurement of oxidative and antioxidant biomarkers
- C (Comparison): Healthy controls
- O (Outcome): Concentrations of oxidative stress and antioxidants biomarkers from patients with OLK and healthy controls

Scientific articles published in scientific databases were selected according to the following inclusion criteria:

- Study design: Observational studies (Cohort, Case-Control studies, and Cross-sectional studies), experimental studies (Randomized and non-randomized clinical trials), studies written in Spanish or English, articles published from 2000 to 2024.
- Patients: Patients with OLK and healthy patients, human adult patients. The diagnosis of oral leukoplakia was based on the exclusion of other oral white lesions and followed the diagnostic criteria established by each original study, using clinical diagnosis with or without histopathological confirmation, depending on the diagnostic approach adopted. Healthy control groups consisted of individuals without clinical signs of oral potentially malignant disorders or other inflammatory oral conditions.
- Intervention: Measurements of oxidative stress compared to antioxidant levels, biomarkers of oxidative stress present in presence of OLK lesion.
- Control subjects: Healthy controls without OLK or other premalignant lesions.
- Outcomes: Measurement of biomarkers, oxidants, and antioxidants, the association between oxidative stress and OLK, oxidative stress as an etiopathogenic factor in OLK.

Studies included patients diagnosed with oral leukoplakia based on clinical criteria, with or without histopathological confirmation, according to the diagnostic approach adopted in each original study. Only original studies reporting oxidative stress–related biomarkers in biological samples from oral leukoplakia patients were included.

Exclusion criteria: We excluded preclinical studies, including animal experiments and *in vitro* studies; systematic reviews; studies not distinguishing OLK from other precancerous lesions; studies before 2000; languages other than English/Spanish; studies with <5 patients; pediatric populations; patients without confirmed OLK diagnosis; HIV or immunosuppressed patients.

A comprehensive literature search was performed in three electronic databases: PubMed/MEDLINE (Medical Literature Analysis and Retrieval System Online), Web of Science and Scopus. The Boolean search algorithms applied in the three databases used the following keywords: “oral leukoplakia”, “OLK”, “potentially malignant disorders”, “oxidative stress”, “redox”, “reactive oxygen species”, “oxidative damage”, “biomarkers” and “antioxidants” (full Boolean strategy available upon request). The last search was performed on January 2025.

For the article selection, the initial screening was done based on the title and study type. After this first selection, screening was conducted by reading the abstracts, and once included, each full article was analyzed based on the oxidative marker measurements, intervention type, sample types assessed, and outcome variables. Study selection was verified by a second reviewer, with final inclusion determined by consensus.

Because the included evidence encompassed several observational study designs, we applied the risk−of−bias tool validated for each specific design to preserve methodological consistency: the Newcastle–Ottawa Scale for case−control and cohort studies, the AXIS tool for cross−sectional studies, and ROBINS−I for non−randomized interventional studies. This approach ensured that each study type was evaluated according to appropriate methodological criteria, despite the heterogeneity of the evidence base.

## 3 Results

**Figure 1:**
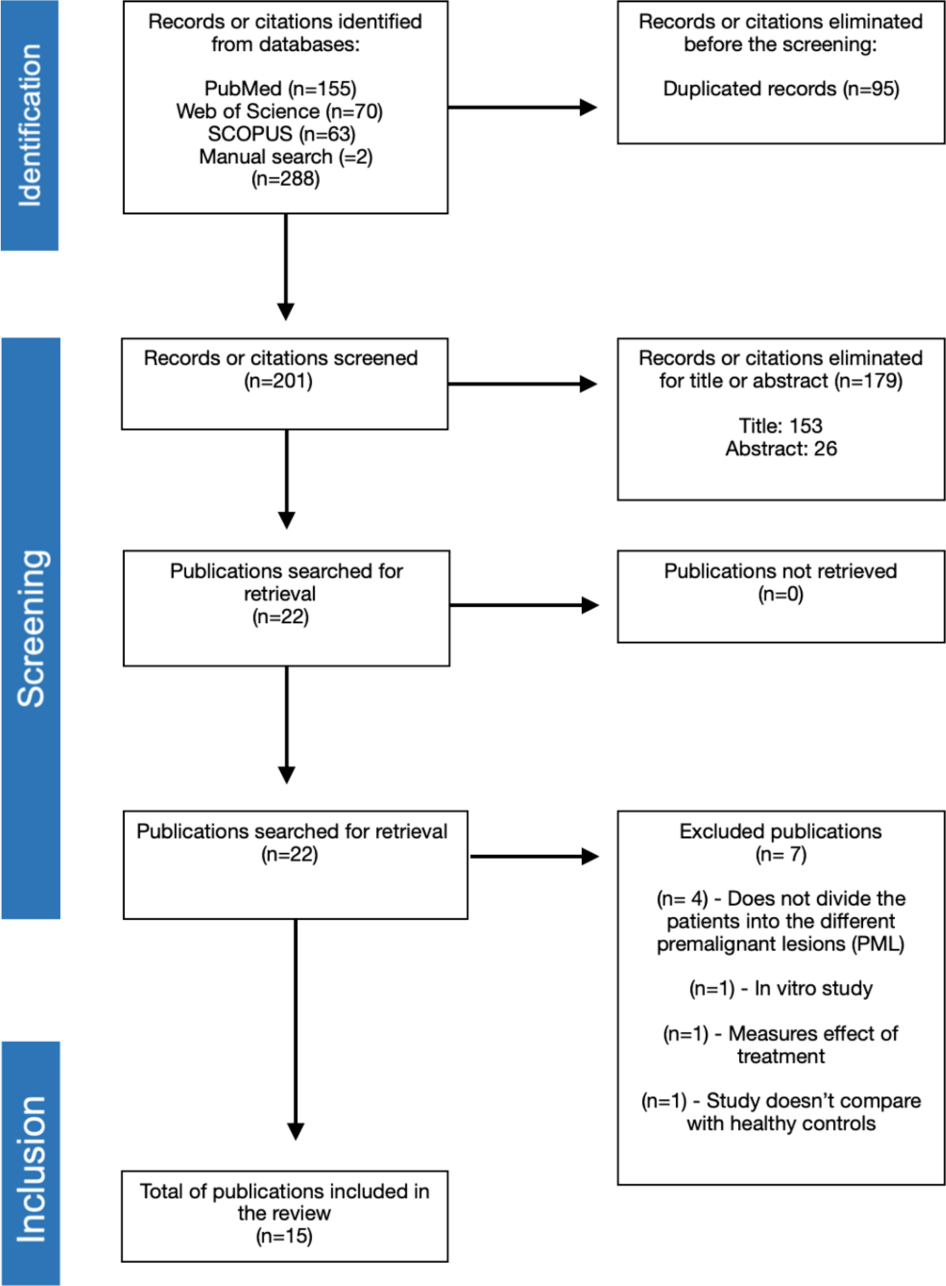
PRISMA flowchart of searching and selection process of titles during systematic review.

**Figure 2:**
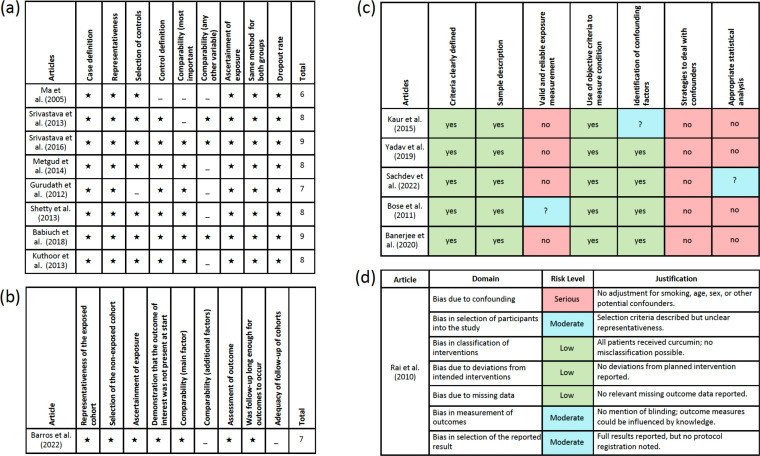
Risk of bias assessment for the included studies using different tools. **(A)** Newcastle-Ottawa Scale for case-control studies; **(B)** Newcastle-Ottawa Scale for cohort studies; **(C)** AXIS tool for cross-sectional studies; **(D)** ROBINS-I tool for non-randomized interventional studies. The star symbol “★” indicates that the criterion was fulfilled, the underscore “_” denotes that the criterion was not met or was not applicable, and the question mark “?” indicates insufficient information or unclear reporting.

A total of 288 articles were obtained from the initial search process: Medline-PubMed (n= 155), Web of Science (n=70), SCOPUS (n= 63) and manual search (n=2). Out of these, 22 were identified as potentially eligible articles through screening by titles and abstracts. The full texts of the identified articles were obtained and evaluated for its eligibility. Consequently, a total of 15 articles met the predetermined inclusion criteria and were ultimately included into this systematic review according to the flowchart of **Figure 1**. The articles were classified based on their risk of bias according to the criteria of **Figure 2**.

Due to substantial heterogeneity in study designs, biomarker types, sample sources, and analytical methods, a quantitative meta−analysis was not feasible.

Regarding the different sample types, of the 15 articles included, 7 used blood or serum samples (Bose et al., 2012; Gurudath et al., 2012; Shetty et al., 2013; Srivastava and Shrivastava, 2016; Yadav et al., 2020; Kuthoor et al., 2022; Sachdev et al., 2022),; 2 used saliva samples (18 and 19); 2 used both saliva and blood samples (20 and 21); and finally, 4 used tissue samples (Ma et al., 2006; Srivastava et al., 2014; Banerjee et al., 2020; Barros et al., 2022). A total of 804 patients were included in the analysis.

Various oxidative stress markers were measured across these different samples. Regarding the antioxidant markers, GSH was the most frequently assessed non-enzymatic antioxidant across the included studies and was evaluated in serum, plasma, saliva, and tissue samples (Bose et al., 2012; Shetty et al., 2013; Metgud and Bajaj, 2014; Srivastava et al., 2014; Srivastava and Shrivastava, 2016; Babiuch et al., 2019; Banerjee et al., 2020; Sachdev et al., 2022). In systemic compartments, including serum, plasma, and erythrocyte fractions, all studies reporting GSH consistently showed lower levels in oral leukoplakia patients compared with healthy controls (Bose et al., 2012; Shetty et al., 2013; Metgud and Bajaj, 2014; Srivastava and Shrivastava, 2016; Sachdev et al., 2022). Similar reductions were observed in salivary simples (Metgud and Bajaj, 2014; Babiuch et al., 2019). In contrast, studies assessing oral tissue reported increased GSH levels in leukoplakia lesions compared to normal oral mucosa. This increase was observed in analyses of whole tissue homogenates as well as in mitochondrial fractions (Srivastava et al., 2014; Banerjee et al., 2020).

Another study further evaluated components of the glutathione redox system, including total glutathione (tGSH), oxidized glutathione (GSSG), and the GSH/GSSG ratio, in salivary simples. The study assessing these markers reported alterations consistent with a more oxidized redox environment in oral leukoplakia patients compared with healthy controls. Specifically, reductions in reduced GSH and in the GSH/GSSG ratio were observed, alongside relative increases in oxidized glutathione (GSSG) or total glutathione, depending on the study (Babiuch et al., 2019).

Other widely studied antioxidant markers include SOD, measured in 7 articles (Gurudath et al., 2012; Srivastava et al., 2014; Srivastava and Shrivastava, 2016; Babiuch et al., 2019; Banerjee et al., 2020; Kuthoor et al., 2022; Sachdev et al., 2022), and GPX, measured in 6 (Ma et al., 2006; Gurudath et al., 2012; Srivastava and Shrivastava, 2016; Babiuch et al., 2019; Banerjee et al., 2020; Sachdev et al., 2022). The enzyme CAT was also examined in 4 articles (Srivastava et al., 2014; Srivastava and Shrivastava, 2016; Banerjee et al., 2020; Sachdev et al., 2022). Most studies reported significantly reduced activities of SOD (Gurudath et al., 2012; Srivastava and Shrivastava, 2016; Kuthoor et al., 2022; Sachdev et al., 2022), CAT (Srivastava and Shrivastava, 2016; Sachdev et al., 2022), and GPx (Gurudath et al., 2012; Srivastava and Shrivastava, 2016; Sachdev et al., 2022) in serum, plasma, or erythrocyte fractions of oral leukoplakia patients compared with controls. Salivary samples also showed reduced antioxidant enzyme activities (Babiuch et al., 2019). Tissue-level findings were more heterogeneous. While SOD and CAT activities were generally reported as reduced in leukoplakia tissue compared to normal mucosa, GPx activity showed variable patterns across tissue-based studies (Srivastava et al., 2014; Banerjee et al., 2020).

Additionally, 6 studies assessed dietary antioxidants, particularly vitamins C and E in serum and salivary samples. All studies assessing these vitamins reported lower levels in oral leukoplakia patients compared with healthy controls (Rai et al., 2010; Bose et al., 2012; Kaur et al., 2016; Sachdev et al., 2022). Trace elements related to antioxidant defense were less frequently assessed. One study evaluating zinc (Zn) reported lower systemic zinc levels in oral leukoplakia patients compared with healthy controls (Bose et al., 2012).

Global antioxidant capacity was assessed using total antioxidant status or capacity (TAS/TAC) in a limited number of studies (Bose et al., 2012; Babiuch et al., 2019). A significant reduction in TAS was observed in plasma samples from oral leukoplakia patients (Bose et al., 2012), while salivary TAC measurements did not show significant differences compared with controls (Babiuch et al., 2019). Uric acid (UA), a major non-enzymatic antioxidant in plasma, was evaluated in a small number of studies. These studies reported lower serum uric acid levels in oral leukoplakia patients compared with healthy controls, although statistical significance and consistency varied across studies (Babiuch et al., 2019; Yadav et al., 2020).

Markers of oxidative damage were consistently altered across biological compartments. Lipid peroxidation markers, including malondialdehyde (MDA) (Rai et al., 2010; Metgud and Bajaj, 2014; Kaur et al., 2016; Babiuch et al., 2019; Sachdev et al., 2022) and thiobarbituric acid reactive substances (TBARS) (Srivastava et al., 2014; Srivastava and Shrivastava, 2016; Banerjee et al., 2020; Sachdev et al., 2022), were elevated in serum, plasma, and salivary samples from oral leukoplakia patients compared with healthy controls in all studies reporting these markers.

Oxidative DNA damage markers, particularly 8-hydroxy-2′-deoxyguanosine (8-OHdG), were assessed in both tissue and salivary samples. Studies evaluating salivary 8-OHdG reported higher levels in oral leukoplakia patients compared with healthy controls (Rai et al., 2010; Kaur et al., 2016; Babiuch et al., 2019). Tissue-based studies further demonstrated increased expression or immunoreactivity of 8-OHdG and 8-nitroguanine in oral leukoplakia lesions compared with normal oral mucosa (Ma et al., 2006; Barros et al., 2022). In the same tissue-based studies, increased iNOS expression was observed alongside enhanced immunoreactivity of nitrative DNA damage markers, including 8-nitroguanine (Ma et al., 2006).

Overall, the included studies demonstrated a clear alteration in redox balance in oral leukoplakia, characterized by reduced systemic and salivary antioxidant defenses, increased oxidative damage markers, and elevated antioxidant levels within lesion tissue. A compartment-specific pattern was consistently observed across different categories of biomarkers.

**Table 1** presents a detailed classification of the data extracted from the included studies, including study design, sample type, and biomarker values. **Table 2** provides a summary of the main findings, highlighting the direction of change of each biomarker across biological compartments. Overall, these findings demonstrate a consistent compartment-specific pattern, characterized by systemic antioxidant depletion and increased oxidative damage, alongside variable or compensatory responses at the tissue level.

**Table 1:** Characteristics of included studies and quantitative assessment of oxidative stress and antioxidant biomarkers in oral leukoplakia (OLK).

Author and year	Sample type	Type of study	N	Antioxidant markers	Oxidant markers
(Bose et al., 2012)	Blood (Plasma)	Cross-sectional study		**GSH:** HC: 10.09±0.89 mg/l; C: 6.09±0.67* mg/l	Not meausred
			HC:23	**TAS:** HC: 2.47±0.43mol/l; C: 1.23±0.45***mol/l	
				**Vit. E:** HC: 10.54±1.1mg/l; C: 5.99±0.82 mg/l**	
			C: 23	**Vit. C:** HC: 1.08±0.16 mg/dl; C: 0.57±0.16 mg/dl**	
				**Zinc:** HC: 91.2±11.8 µg/dl; C: 59.9±6.91 µg/dl***	
(Gurudath et al., 2012)	Blood (RBC)	Case-control study	HC: 25	**SOD:** HC: 199.35 U/ml; C: 91.52 U/ml ***	Not measured
			C:25	**GPx:** HC: 60.46* U/g Hb; C: 21.55 U/g Hb***	
(Shetty et al., 2013)	Blood (Serum)	Case-control study	HC: 25	**GSH:** HC: 1.88 ± 0.36 mmol/l; C: 01.04 ± 0.22 mmol/l**	Not meausred
			C:25		
(Srivastava and Shrivastava, 2016)	Blood (Plasma+RBC)	Case-control study	HC: 20	**GSH:** HC: 51.10±2.09 mg/dl; C: 40.15±3.09 mg/dl**	**TBARS:** HC: 1.30±0.40 nM/ml; C: 2.20±0.44 nM/ml**
				**SOD:** HC: 4.70±1.26 U/g Hb; C: 2.09±0.08 U/g Hb*	
			C:20	**CAT:** HC: 3.46±0.85 U/g Hb; C: 1.37±0.08 U/g Hb**	
				**GPx:** HC: 25.07±1.55 U/g Hb; C: 19.09±0.56 U/g Hb**	
(Yadav et al., 2020)	Blood (Serum)	Cross-sectional study	HC: 30	**UA:** HC: 5.16± 0.98 mg/dl; C: 3.79±1.23mg/dl	Not measured
			C:25		
(Sachdev et al., 2022)	Blood (Plasma+RBC+Serum)	Cross-sectional study	HC: 70	**GSH:** HC: 13.24±0.94 mg/dl; C: 2.02±0.322 mg/dl**	**MDA:** HC: 1.96±0.145 nmol/ml; C: 5.68±0.322 nmol/ml***
				**SOD:** HC: 233.64±11.89 U/100 mg protein; C: 188.45±8.54 U/100 mg protein***	
				**CAT:** HC: 35.3±3.11 U/mg protein; 13.51±2.32 U/mg protein**	
			C: 70	**GPx:** HC: 15.23±2.68 U/mg protein; C: 2.67±1.34 U/mg protein**	**LOOH:** HC: 276.46±17.66 µmol/ml; C: 467.65±17.43 µmol/ml***
				**Vit. E:** HC: 11.74±0.566 mg/l; C: 0.73±0.211 mg/l***	
				**Vit. C:** HC: 2.78±0.31 mg/dl; C: 0.41±0.162 mg/dl**	
(Kuthoor et al., 2022)	Blood (Plasma)	Case-control study	HC:25	**SOD:** HC: 0.074±0.014 U/ml; C: 0.052±0.012 U/ml***	Not meausred
			C: 29		
(Kaur et al., 2016)	Saliva	Cross-sectional study	HC:40	**Vit. E:** HC: 1.4 ± 0.6 μg/l; C: 0.57 ± 0.16 μg/l**	**MDA:** HC: 0.08± 0.07 μmol/l; C: 0.33 ± 0.07 μmol/l**
			C:40	**Vit. C:** HC: 1.2 ± 0.6 μg/l; C: 0.55 ± 0.13 μg/l**	**8-OHdG:** HC: 0.07± 0.07ng/ml; C: 0.36 ± 0.07ng/ml**
(Babiuch et al., 2019)	Saliva	Case-control study (pilot study)	HC: 20	**GSH:** HC: 0.02 ±0.01μmol/l; C: 0.01± 0.02μmol/l***	**MDA:** HC: 2.32 ± 5.36μmol/l: C: 8.30 ± 14.22 μmol/l
				**tGSH:** HC: 0.16 ±0.25μmol/l; C: 0.18± 0.27μmol/l	
				**GSSH:** HC: 0.23 ± 0.22 μmol/l; C: 0.26 ± 0.25 μmol/l	
				**GSH/GSSG:** HC: 0.27± 0.43; C: 0.21±0.64**	
				**SOD:** HC: 2.36 ± 2.42 U/ml; C: 3.40 ± 3.92 U/ml**	
			C:20	**GPx:** HC: 90.60 ±18.65U/l; C: 81.34 ±22.56U/l	**8-OHdG:** HC: 8.58± 4.59ng/ml; C: 11.54 ± 8.22ng/ml
				**TAC:** HC: 0.51± 0.34 mmol/l; C: 0.74 ± 0.44 mmol/l	
				**GR:** C: 17.7 ± 27.48 U/l: HC: 7.68 ±6.47 U/l	
				**UA**: HC: 256.79 ±185.20 μmol/l; C: 386.36 ±235.96μmol/l	
(Rai et al., 2010)	Saliva and blood (serum)	Pre-post interventional study	HC:25	**BLOOD:**	**BLOOD:**
				**Vit. E:** HC: 8.97± 2.34 μg/l; C: 8.01± 1.23 μg/l***	**MDA:** HC: 0.98 ± 0.86μmol/l; C:1.23 ± 0.56 μmol/l**
				**Vit. C:** HC: 9.05± 2.21 μg/l; C: 8.78 ± 3.12 μg/l***	**8-OHdG:** HC: 2.17 ± 1.45 ng/ml; C: 2.13 ± 1.12 ng/ml***
			C:25	**SALIVA:**	**SALIVA:**
				**Vit. E:** HC: 0.91 ± 0.43 μg/l; C: 0.65 ± 0.31 μg/l***	**MDA:** HC: 0.11 ± 0.12 μmol/l; C: 0.36± 0.17 μmol/l***
				**Vit. C:** HC: 1.46 ±0.86 μg/l; C: 1.08 1± 0.98 μg/l***	**8-OHdG:** HC: 0.11 ± 0.12 ng/ml; C: 0.34 ± 0.24 ng/ml
(Metgud and Bajaj, 2014)	Saliva and blood (serum)	Case-control study	HC: 20	**BLOOD:**	**BLOOD:**
				**GSH:** HC: 32.18 ± 5.53nmol/dl; C: 21.47 ± 3.35 nmol/dl**	**MDA:** HC: 2.93 ± 0.79nmol/dl; C: 3.31 ± 0.41nmol/dl*
			C: 30	**SALIVA:**	**SALIVA:**
				**GSH:** HC: 9.74± 0.53 nmol/dl; C: 8.67 ± 1.20 nmol/dl**	**MDA:** HC: 19.98 ± 0.81nmol/dl; C: 20.97 ± 1.23 nmol/dl*
(Ma et al., 2006)	Tissue	Case-control study	HC:4 C:19	Not meausred	**8-OHdG:** HC: Negative (IR); C: Strongly positive (IR)**
					**8-nitroguanine:** HC: Negative (IR); C: Strongly positive (IR)**
					**iNOS:** HC: Negative (IR); C: Strongly positive (IR)**
(Srivastava et al., 2014)	Tissue	Case-control study	HC: 20	**GSH:** HC: 22.9±1.10 mg/dl; C: 30.43±2.9 mg/dl***	**TBARS:** HC: 127.93 ± 2.97 nM/ml; C: 91.99 ± 2.97 nM/ml***
				**SOD:** HC: 18.54 ± 0.54; C: 14.48 ± 1.05***	
			C: 20	**GPx:** HC: 15.16 ± 0.48 U/g Hb; C: 22.99 ± 3.43 U/g Hb***	
				**CAT:** HC: 10.46 ± 0.79 U/g Hb; C: 6.36 ± 1.10 U/g Hb***	
(Banerjee et al., 2020)	Tissue (mitochondria)	Cross-sectional study	HC: 20	**GSH:** HC: 11.3 ± 0.716 mM; C: 30.43±2.9 mM	**Lipid peroxide:** HC: 1.57 ± 0.286 nmol/mg of protein; C: 2.02 ± 0.16 nmol/mg of protein
				**SOD:** data not shown	
			C: 12	**CAT:** HC: 6.4 ± 0.268 U/mg/min; C: 3.1 ± 0.156 U/mg/min	
				**GPx:** data not shown	
(Barros et al., 2022)	Tissue	Prospective longitudinal study	HC: 10	Not meausred	**8-OHdG:** HC: Negative (IR in cytoplasm); C: Strongly positive (IR in cytoplasm)*
			C: 44		

* indicates statistically significant results in all included studies for the corresponding marker and compartment.

**Table 2:** Direction and consistency of oxidative stress and antioxidant biomarker alterations across biological compartments in oral leukoplakia.

Marker	Blood	Saliva	Tissue	References
Non-enzymatic antioxidants and related markers				
GSH	↓* (n=4)	↓* (n=2)	↑ (n=2)	Blood: Bose et al.,2012; Shetty et al., 2013; Srivastava and Shrivastava, 2016; Sachdev et al., 2022
				Saliva: Babiuch et al., 2019; Rai et al., 2010
				Tissue: Srivastava et al., 2014; Banerjee et al., 2020
tGSH	—	↑ (n=1)	—	Babiuch et al., 2019
GSSG	—	↓ (n=1)	—	Babiuch et al., 2019
GSH/GSSG ratio	—	↓* (n=1)	—	Babiuch et al., 2019
Uric acid (UA)	↓ (n=1)	↓ (n=1)	—	Blood: Yadav et al., 2020
				Saliva: Babiuch et al., 2019
Zinc (Zn)	↓* (n=1)	—	—	Bose et al., 2012
Enzymatic antioxidants				
SOD	↓* (n=4)	↓* (n=1)	↓ (n=2)	Blood: Gurudath et al., 2012; Srivastava and Shrivastava, 2016; Sachdev et al., 2022; Kuthoor et al., 2022
				Saliva: Babiuch et al., 2019
				Tissue: Srivastava et al., 2014; Banerjee et al., 2020
CAT	↓* (n=2)	—	↓ (n=2)	Blood: Srivastava and Shrivastava, 2016; Sachdev et al., 2022
				Tissue: Srivastava et al., 2014; Banerjee et al., 2020
GPx	↓* (n=3)	↓ (n=1)	Variable (n=2)	Blood: Gurudath et al., 2012; Srivastava and Shrivastava, 2016; Sachdev et al., 2022
				Saliva: Babiuch et al., 2019
				Tissue: Srivastava et al., 2014; Banerjee et al., 2020
Dietary antioxidants and global antioxidant capacity				
Vitamin C	↓* (n=3)	↓* (n=2)	—	Blood: Bose et al., 2012; Sachdev et al., 2022; Rai et al., 2010
				Saliva: Kaur et al., 2016; Rai et al., 2010
Vitamin E	↓* (n=3)	↓* (n=2)	—	Blood: Bose et al., 2012; Sachdev et al., 2022; Rai et al., 2010
				Saliva: Kaur et al., 2016; Rai et al., 2010
TAS/TAC	↓* (TAS, n=1)	↓ (TAC, n=1)	—	Blood: Bose et al., 2012
				Saliva: Babiuch et al., 2019
Oxidative and nitrative damage markers				
MDA	↑* (n=2)	↑ (n=4)	—	Blood: Sachdev et al., 2022; Metgud and Bajaj, 2014
				Saliva: Kaur et al., 2016; Babiuch et al., 2019; Rai et al., 2010; Metgud and Bajaj, 2014
TBARS	↑* (n=1)	—	↓* (n=1)	Blood: Srivastava and Shrivastava, 2016
				Tissue: Srivastava et al., 2014
Lipid peroxide/LOOH	↑* (LOOH, n=1)	—	↑ (Lipid peroxide, n=1)	Blood: Sachdev et al., 2022
				Tissue: Banerjee et al., 2020
8-OHdG	↑* (n=1)	↑ (n=3)	↑* (n=2)	Blood: Rai et al., 2010
				Saliva: Kaur et al., 2016; Babiuch et al., 2019; Rai et al., 2010; Metgud and Bajaj, 2014
				Tissue: Ma et al., 2006; Barros et al., 2022
8-Nitroguanine	—	—	↑* (n=1)	Ma et al., 2006
iNOS	—	—	↑* (n=1)	Ma et al., 2006

*Indicates statistically significant results in all included studies for the corresponding marker and compartment.↑: Increase; ↓: Decrease.

## 4 Discussion

### 4.1 Overall redox imbalance in oral leukoplakia

This systematic review identifies a consistent pattern of oxidative imbalance in oral leukoplakia, characterized by reduced antioxidant defenses and increased oxidative damage at the systemic and salivary levels, alongside increased antioxidant concentrations within lesion tissue.

### 4.2 Compartment-specific redox responses

GSH stands out as the most consistently investigated biomarker across the studies included in this review, underscoring its pivotal role in reflecting oxidative stress and redox imbalance in OLK. GSH is the primary and most abundant antioxidant within cells and serves a vital function in protecting the organism from damage and disease, playing a key role in redox signaling and regulating cell proliferation, apoptosis, and immune function (Sies, 1999; Lu, 2013; Yoshida et al., 2019). Various studies have reported alterations in normal GSH levels in a variety of diseases, illustrating the broader relevance of GSH dysregulation in chronic disease processes involving oxidative stress (Townsend et al., 2003; Asher and Guilford, 2016; Senghore et al., 2018; Lana et al., 2024).

In OLK specifically, it exists in cells primarily in two forms: the reduced form (GSH) and the oxidized form (GSSG). Together, these make up total glutathione (tGSH), which reflects the overall availability of glutathione in the system. The GSH/GSSG ratio is commonly used to assess the cellular redox status, as it indicates the balance between antioxidant capacity and oxidative burden. These three measures are closely related and provide complementary information about the redox environment within cells (Lu, 2013; Yoshida et al., 2019).

Consistently, serum and saliva samples showed reduced GSH levels, compatible with systemic depletion under sustained oxidative load. Although GSH is predominantly an intracellular antioxidant and extracellular concentrations are considerably lower, salivary and serum GSH may still indirectly reflect systemic redox imbalance rather than local antioxidant capacity per se. In contrast, increased GSH levels within OLK lesions point to a compartment-specific adaptive antioxidant response at the tissue level (Bose et al., 2012; Shetty et al., 2013; Metgud and Bajaj, 2014; Srivastava et al., 2014; Babiuch et al., 2019; Banerjee et al., 2020; Sachdev et al., 2022). An important role of GSH is detoxification of chemical carcinogens and protecting the cells against the cytotoxic ROS. Carcinogens from tobacco smoke and quid are primarily detoxified by GSH dependent enzymes. Continuous exposure of the oral mucosa to carcinogenic agents results in their gradual accumulation within the surrounding tissues, which in turn enhances GSH expression in tumor sites (Srivastava et al., 2014). Evidently, it is important for prevention of oral cancer appearance, as GSH detoxifies carcinogens and lipid peroxidation products while supporting immune function (Metgud and Bajaj, 2014). The GSH/GSSG ratio findings in saliva are compatible with a more oxidized extracellular milieu, reinforcing the notion of compartmentalized redox responses (Lu, 2013; Babiuch et al., 2019; Yoshida et al., 2019).

### 4.3 Glutathione system and redox regulation

The antioxidant enzyme glutathione reductase (GR) is closely linked to the GSH system, as it catalyzes the NADPH-dependent reduction of GSSG back to its active reduced form, GSH (Couto et al., 2016). Under oxidative stress, intracellular levels of GSSG increase as GSH is consumed by enzymes such as GPx in the detoxification of ROS (Srivastava et al., 2014; Couto et al., 2016; Babiuch et al., 2019). Without sufficient GR activity, GSH cannot be efficiently regenerated, and the redox balance shifts toward a more oxidized state (Couto et al., 2016; Babiuch et al., 2019). GR therefore plays a central role in preserving redox homeostasis and supporting antioxidant defense. Although evidence is limited, trends toward higher GR in potentially malignant lesions did not reach statistical significance, and should be interpreted cautiously (Babiuch et al., 2019).

### 4.4 Enzymatic antioxidant defenses

GPx is a family of eight antioxidant enzymes, GPx1-GPx8, that use GSH to neutralize lipid peroxides (Pei et al., 2023). Different studies reviewed assessed the general expression of this enzyme, but also GPx1 and GPx4 were assessed specifically. While GPx1 is abundantly and widely expressed across tissues and primarily targets H2O2 and small peroxides, GPx4 is unique in its ability to directly reduce complex lipid hydroperoxides within membranes. This makes GPx4 essential for preventing ferroptosis, a form of regulated cell death driven by lipid peroxidation. Their dysfunction contributes to oxidative stress-related damage and is implicated in cancer, inflammation, and other pathologies (Xie et al., 2023). GPX1 shows dual roles in cancer biology, acting both as a tumor suppressor and promoter depending on context, and influences processes such as cell proliferation, apoptosis, and therapy resistance (Zhao et al., 2022). Across the included studies, systemic GPx activity tended to be lower in OLK than in controls, whereas tissue data were more heterogeneous, with reports of higher GPx in lesions in at least one series (Gurudath et al., 2012; Srivastava et al., 2014; Srivastava and Shrivastava, 2016; Babiuch et al., 2019; Sachdev et al., 2022).

### 4.5 Lipid peroxidation and oxidative damage markers

Lipid peroxidation refers to the oxidative degradation of lipids, initiated when ROS attack polyunsaturated fatty acids in cell membranes (Moore and Roberts, 1998; Tsikas, 2017). This peroxidation cascade leads to structural damage of the cell membrane integrity and contributes to cellular dysfunction. This process generates various reactive compounds, among which MDA is one of the most abundant and widely studied (Moselhy et al., 2013; Ayala et al., 2014). MDA can be measured directly, or indirectly through TBARS assay, in which it is the primary detectable product (Moore and Roberts, 1998; Tsikas, 2017). Together, measurements of lipid peroxidation, TBARS, and MDA provide complementary insight into oxidative membrane damage and the extent of redox imbalance in tissue or body fluids (Grotto et al., 2009; Moselhy et al., 2013).

In OLK, salivary and systemic MDA and TBARS were generally higher than in healthy controls, aligning with increased oxidative damage (Rai et al., 2010; Metgud and Bajaj, 2014; Kaur et al., 2016; Srivastava and Shrivastava, 2016; Babiuch et al., 2019; Sachdev et al., 2022). By contrast, tissue lipid peroxidation markers showed mixed results, significantly lower in one tissue series (Srivastava et al., 2014) versus higher mitochondrial lipid peroxides in another (Banerjee et al., 2020), highlighting possible differences in assay targets, lesion stage, and sampling depth. These discrepancies may reflect biological compartmentalization and methodological heterogeneity rather than a uniform suppression of lipid peroxidation within the lesion (Moore and Roberts, 1998; Ayala et al., 2014; Xiao et al., 2024).

Another of the key antioxidant enzymes, SOD, catalyzes the dismutation of superoxide anions (O_2_•^-^) into H2O2, thereby converting reactive species into less harmful non-radical products, which are then further neutralized by CAT, among others. Through this mechanism, these enzymes help preventing O_2_•^-^-induced lipid peroxidation and protects cellular components from oxidative damage (Rasheed, 2024). Several studies have shown that salivary, serum and tissue SOD and CAT levels are significantly reduced in patients with OLK and oral cancer compared to healthy controls, which is consistent with consumption or down−regulation under sustained oxidative pressure; however, some salivary datasets showed divergent SOD activity, cautioning against over−generalization (Gurudath et al., 2012; Srivastava et al., 2014; Srivastava and Shrivastava, 2016; Babiuch et al., 2019; Banerjee et al., 2020; Kuthoor et al., 2022; Sachdev et al., 2022). This suggests a systemic impairment of antioxidant defenses and supports the role of these enzymes in protecting against DNA damage in the extracellular microenvironment.

### 4.6 Non-enzymatic antioxidants

Numerous studies have also investigated the concentrations of non-enzymatic antioxidants, including uric acid (UA) and vitamins E and C. UA, despite being a major antioxidant in the human plasma, has a dual role as an antioxidant (primarily in plasma) or pro-oxidant (primarily within the cell), one explanation for this paradox could be that a rise in uric acid represents an attempted protective response by the host (Sautin and Johnson, 2008). Furthermore, UA levels are influenced by several external factors, including alcohol consumption and diet, and studies have shown that both alcohol and tobacco can have an impact on salivary UA levels (Gherghina et al., 2022; Wen et al., 2024). The reviewed studies comparing serum and saliva levels of UA in patients with OLK and healthy controls showed minor and inconsistent differences (slightly lower in serum, not significant, and higher in saliva in a pilot series) limiting its clinical utility as a standalone marker (Babiuch et al., 2019; Yadav et al., 2020). Thus, its dual antioxidant/pro-oxidant role and sensitivity to dietary and lifestyle factors make it less reliable as a clinical marker in this context.

Vitamin E, a fat-soluble antioxidant, mainly protects cell membranes by preventing the formation of reactive oxygen species during fat oxidation, inhibiting lipid peroxidation and the spread of free radical reactions (Lewis et al., 2019) Vitamin C (ascorbic acid, or ascorbate), a potent water-soluble antioxidant, is essential for preventing and treating scurvy, with roles in tissue repair, collagen formation, enzymatic production, and survival. It is a water-soluble electron donor that participates in regenerating other antioxidants and directly neutralizes ROS (Chambial et al., 2013; Andrés et al., 2024). Patients with OLK and OSCC, showed significantly lower levels of vitamins C and E in saliva compared to healthy controls, with further reduction in more advanced disease stages (Rai et al., 2010; Bose et al., 2012; Kaur et al., 2016; Sachdev et al., 2022). This supports the theory that free radicals consume the body’s antioxidant reserves in the progression of premalignant conditions. At the same time, a non−randomized interventional study showed that treatment with curcumin increased levels of vitamins C and E, as well as reducing lipid peroxidation and DNA damage, suggesting that levels can also be altered therapeutically (Rai et al., 2010).

### 4.7 Oxidative DNA damage and carcinogenic pathways

In addition to the enzymatic and thiol-based redox markers discussed previously, several studies have highlighted the role of oxidative DNA damage markers, specifically 8-OHdG and 8-nitroguanine, as indicators of genotoxic stress in OLK and its potential malignant transformation. These DNA lesions arise from oxidative and nitrative stress, respectively, and are found elevated in various human cancers (Murata, 2018; Arfin et al., 2021; Omari Shekaftik and Nasirzadeh, 2021). DNA damage markers such as 8-OHdG and 8-nitroguanine were elevated in tissue and saliva, with increased staining in dysplastic areas and strong immunoreactivity observed in OLK lesions (Ma et al., 2006; Rai et al., 2010; Kaur et al., 2016; Babiuch et al., 2019; Barros et al., 2022). A correlation between dysplasia severity and cytoplasmic 8-OHdG expression has been reported, suggesting its relevance in disease progression (Barros et al., 2022). In particular, cytoplasmic accumulation of 8-OHdG has been linked to mitochondrial DNA damage, which could play a role in early malignant transformation by altering cellular energy metabolism (Barros et al., 2022). Elevated levels of both 8-OHdG and 8-nitroguanine have been observed in oral epithelial tissue from OLK patients with colocalization. This colocalization and the parallel increase in iNOS expression suggest that nitrative stress plays a mechanistic role in early oral carcinogenesis, likely mediated by ONOO^-^ generation and subsequent DNA base modification (Ma et al., 2006). Interestingly, intervention with curcumin resulted in a reduction of 8-OHdG levels in both saliva and serum, along with clinical improvement in patients with OLK (Rai et al., 2010). This indicates the potential therapeutic responsiveness of this biomarker, further supporting its value in monitoring disease activity.

### 4.8 Global antioxidant capacity measures

Total antioxidant status (TAS) and total antioxidant capacity (TAC) are two terms that are often used interchangeably in the literature, as both refer to the overall antioxidant capacity of the body. Both biomarkers provide an integrated measure of the interaction between known and unknown antioxidants in the body, reflecting the balance between oxidants and antioxidants (Fraga et al., 2014). One of the reviewed studies showed significantly reduced levels of TAS in OLK patients compared to healthy controls, suggesting an impaired antioxidant defense mechanism in the pathogenesis of the condition (Bose et al., 2012). Another study showed lower levels of TAC values in patients with OLK, although these differences were not statistically significant (Babiuch et al., 2019). Methodological diversity in TAC/TAS assays and the influence of diet and systemic conditions should be considered when interpreting these measures (Fraga et al., 2014).

### 4.9 Influence of lifestyle-related oxidative stressors

Carcinogens such as tobacco and alcohol have been shown as strong contributors to oxidative stress in OLK, with studies linking them to increased MDA and reduced antioxidant levels (Metgud and Bajaj, 2014; Srivastava et al., 2014; Srivastava and Shrivastava, 2016; Gherghina et al., 2022). The chronic exposure to carcinogens induces persistent ROS generation, which not only damages cellular structures but also depletes antioxidant systems like GSH and CAT, creating an environment conducive to malignant transformation (Metgud and Bajaj, 2014; Srivastava et al., 2014). Recent evidence further supports this concept, showing that cigarette smoke promotes cellular senescence and a pro-inflammatory secretory phenotype in oral mucosa, reinforcing its role in early carcinogenic processes (Pérez-Leal et al., 2026). Accordingly, future studies should stratify by tobacco/alcohol exposure and adjust for these confounders in multivariable analyses to disentangle lesion-specific redox changes from lifestyle-related effects.

### 4.10 Limitations

This review has some limitations that should be considered when interpreting the findings. First, the included studies exhibit marked methodological heterogeneity, encompassing cross−sectional, case−control, cohort, and non-randomized interventional designs, which precluded direct comparison and prevented quantitative synthesis. Second, assay variability and inconsistent reporting of biomarker units further limited standardization. Third, most studies did not adequately control for major confounders such as tobacco use, alcohol consumption, diet, systemic conditions, or medication, all of which can significantly influence oxidative stress markers. Additionally, the predominance of cross-sectional evidence restricts causal interpretation, and sample sizes were generally small. Taken together, these limitations highlight the need for well-designed, longitudinal studies with standardized biomarker protocols and appropriate adjustment for lifestyle and clinical variables.

## 5 Conclusion

The available evidence indicates a consistent pattern of redox imbalance in oral leukoplakia, characterized by increased oxidative damage and reduced systemic antioxidant capacity, with a possible compensatory increase in tissue-based glutathione defenses. Salivary and systemic markers such as MDA and 8−OHdG show reproducible differences between OLK patients and healthy controls, suggesting their potential utility as indicators of oxidative imbalance. However, the heterogeneity of study designs, lack of confounder control, and absence of longitudinal data prevent any conclusions regarding diagnostic accuracy or prognostic value. Future research should employ standardized analytical protocols, adjust for lifestyle-related oxidative stressors, and incorporate prospective designs to better determine the clinical relevance of these biomarkers.

## Data Availability

The original contributions presented in the study are included in the article. Further inquiries can be directed to the corresponding authors under reasonable request.
